# Transcriptome and Phenotype Integrated Analysis Identifies Genes Controlling Ginsenoside Rb1 Biosynthesis and Reveals Their Interactions in the Process in *Panax ginseng*

**DOI:** 10.3390/ijms232214016

**Published:** 2022-11-13

**Authors:** Yue Jiang, Sizhang Liu, Li Li, Kaiyou Zang, Yanfang Wang, Mingzhu Zhao, Kangyu Wang, Lei Zhu, Ping Chen, Jun Lei, Yi Wang, Meiping Zhang

**Affiliations:** 1 College of Life Science, Jilin Agricultural University, Changchun 130118, China; 2 College of Chinese Medicinal Materials, Jilin Agricultural University, Changchun 130118, China; 3 Research Center for Ginseng Genetic Resources Development and Utilization, Jilin Agricultural University, Changchun 130118, China

**Keywords:** gene identification, quantitative trait, gene for Rb1 biosynthesis, biomarker, gene interaction, ginseng

## Abstract

Genes are the keys to deciphering the molecular mechanism underlying a biological trait and designing approaches desirable for plant genetic improvement. Ginseng is an important medicinal herb in which ginsenosides have been shown to be the major bioactive component; however, only a few genes involved in ginsenoside biosynthesis have been cloned through orthologue analysis. Here, we report the identification of 21 genes controlling Rb1 biosynthesis by stepwise ginseng transcriptome and Rb1 content integrated analysis. We first identified the candidate genes for Rb1 biosynthesis by integrated analysis of genes with the trait from four aspects, including gene transcript differential expression between highest- and lowest-Rb1 content cultivars, gene transcript expression–Rb1 content correlation, and biological impacts of gene mutations on Rb1 content, followed by the gene transcript co-expression network. Twenty-two candidate genes were identified, of which 21 were functionally validated for Rb1 biosynthesis by gene regulation, genetic transformation, and mutation analysis. These genes were strongly correlated in expression with the previously cloned genes encoding key enzymes for Rb1 biosynthesis. Based on the correlations, a pathway for Rb1 biosynthesis was deduced to indicate the roles of the genes in Rb1 biosynthesis. Moreover, the genes formed a strong co-expression network with the previously cloned Rb1 biosynthesis genes, and the variation in the network was associated with the variation in the Rb1 content. These results indicate that Rb1 biosynthesis is a process of correlative interactions among Rb1 biosynthesis genes. Therefore, this study provides new knowledge, 21 new genes, and 96 biomarkers for Rb1 biosynthesis useful for enhanced research and breeding in ginseng.

## 1. Introduction

Genes are the keys to deciphering the molecular mechanism underpinning a biological trait and designing approaches efficient for plant and animal genetic improvement. Therefore, several methods have been developed to clone the genes controlling a biological trait, such as map-based cloning [1] and mutagenesis [2,3]. These methods include two parts: candidate gene identification and gene function validation. QTL mapping, eQTL mapping, gene differential expression (DE) analysis, and genome-wide association study (GWAS) have been widely used for the genome-wide identification of candidate genes controlling a biological trait, while genetic transformation, RNA interference (RNAi), gene editing, and gene regulation have been widely used to validate the functions of the candidate genes. Therefore, candidate gene identification has been the prerequisite for gene cloning because most genes are known only by phenotypes. These methods have greatly contributed to the cloning of genes controlling qualitative and quantitative traits [1]. However, only a few of the genes have been cloned for each of the quantitative traits to date [4]. Since most traits of agricultural or biological importance are quantitative traits controlled by numerous genes, the limited number of genes cloned to date for each of the traits is insufficient not only for comprehensively deciphering the molecular mechanisms of the traits but also for designing approaches efficient for the manipulation of the traits in agriculture. Therefore, it is necessary to develop a procedure efficient for genome-wide identification of candidate genes controlling a complex trait to accelerate the process of gene cloning and promote functional genomics research.

The aim of the present study was to develop a method that is accurate, efficient, readily usable, and widely applicable for the genome-wide identification of the candidate genes controlling a complex trait using ginseng ginsenoside Rb1 as the target trait. Ginseng (*Panax ginseng* Mey.), abundant in numerous bioactive components, especially ginsenosides, is grown in Asia and North America for health food, health product, and medicine. Ginseng is a tetraploid perennial that has a large complex genome, with a genome size of approximately 3.3 Gb. Although three genomes of ginseng have been sequenced [5,6,7], and a massive number of transcriptomes have been developed from different genotypes of ginseng [8], different developmental stages [9,10], different plant parts [10,11], and different treatment conditions [12], no artificial mapping population has been developed, and no genetic map has been available, as has been for annual crops such as rice, maize, wheat, soybean, and cotton. Ginseng genome research remains largely under-studied compared with those of annual crops. If a procedure that is efficient for the genome-wide identification of the candidate genes controlling a complex trait is developed in ginseng, it will be applicable to other crops whose genome research has been far more advanced. Importantly, a desirable method has been developed and widely used in ginseng to validate the functions of candidate genes involved in ginsenoside biosynthesis [8,13,14,15,16,17,18], which will facilitate the validation of the candidate genes controlling the target trait. Ginsenoside Rb1 is one of the most important ginsenosides [19], and the biosynthesis of Rb1 has been shown to be a complex biological process controlled by numerous genes.

The present study analyzed the transcriptomes of four-year-old plant roots of 42 cultivars selected from a ginseng GWAS panel against their Rb1 contents. The analysis was performed from four aspects, including gene transcript differential expressions between cultivars with highest- and lowest-Rb1 contents, the correlation between gene transcript expression and Rb1 content among the cultivars, the biological impact of gene SNP mutation on Rb1 content, and the co-expression network of genes with the previously cloned genes that were involved in ginsenoside biosynthesis [18,20,21,22,23,24,25,26,27]. The major advances of this analysis procedure over the existing integrated analysis of transcriptomes with the target trait for candidate identification included three aspects: (1) the use of transcript expressions for gene differential expression analysis, correlation analysis, and gene co-expression network analysis; (2) the use of gene transcript expression–target trait correlation in a population; and (3) the use of the impact of SNP or InDel mutation contained in the gene on the target trait. Given that different transcripts spliced from the same gene may have different biological functions [4], the candidate genes identified are more accurate than those identified based on genes when the gene transcripts are used for candidate gene identification. We identified 22 candidate genes for Rb1 biosynthesis, the functions of 21 of which in Rb1 biosynthesis were validated. Using the Rb1 biosynthesis genes, we, for the first time, developed 96 biomarkers for Rb1 biosynthesis and studied the gene interactions underlying Rb1 biosynthesis at the genome level. Therefore, this study has identified 21 new genes and 96 biomarkers for Rb1 biosynthesis and substantially advanced our understanding of the molecular mechanisms underlying Rb1 biosynthesis.

## 2. Results

### 2.1. Variability of Rb1 Content in Four-Year-Old Plant Roots among Cultivars

**Figure 1:**
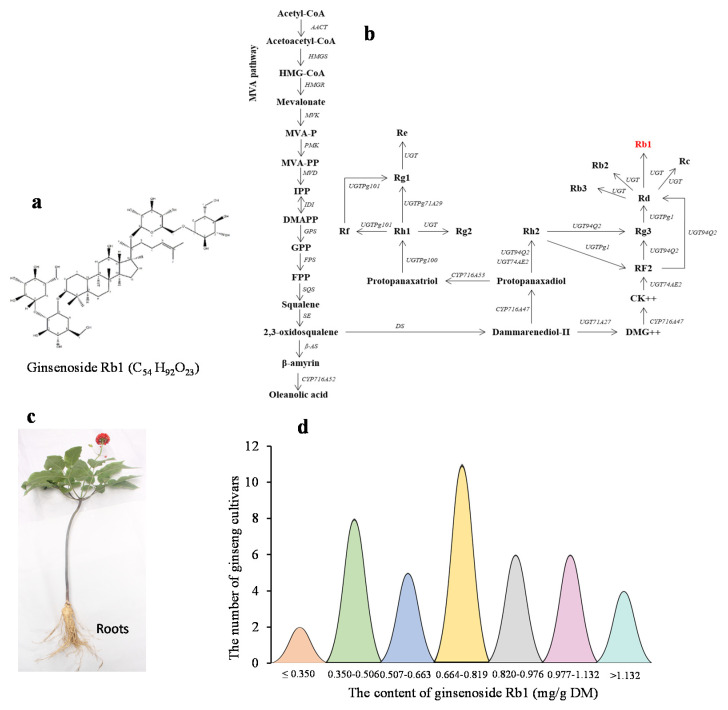
Variation of ginsenoside Rb1 content in the four-year-old plant roots of ginseng cultivars. (**a**) Ginsenoside, Rb1, and its chemical structure. (**b**) The position of Rb1 in the ginsenoside biosynthesis pathway (modified from [28]). (**c**) A four-year-old ginseng plant used for determining the content of Rb1 in roots. (**d**) Variation of Rb1 content in four-year-old ginseng plant roots among cultivars selected from a ginseng genome-wide association study (GWAS) panel. DM, dry matter.

Ginsenoside, Rb1, is a secondary metabolite with a chemical formula of C_54_H_92_O_23_ (Figure 1a). Figure 1b shows the position of Rb1 in the ginsenoside biosynthesis pathway [28]. The root is the major organ where ginsenosides are stored, thus being the major economic produce of ginseng production. The contents of Rb1 in the roots of four-year-old plants (Figure 1c) were quite high and varied dramatically among cultivars, with an average content of 0.75 mg/g root dry matter (DM), a coefficient of variance (CV) of 36.86%, and an arrange of 3.57-fold from 0.39 mg/g root DM to 1.38 mg/g root DM. The variation in the Rb1 contents of the four-year-old plant roots was nearly normally distributed among the cultivars (Figure 1d). These results confirmed that the Rb1 content was quantitatively inherited and controlled by multiple genes.

### 2.2. Genome-Wide Identification of PgRb1 Candidate Genes for Rb1 Biosynthesis

We genome-wide identified the *PgRb1* biosynthesis candidate genes using a sequential procedure of four steps. First, we conducted differential expression analysis of the gene transcripts expressed in the four-year-old plant roots between the 28 cultivars that had the highest (14 cultivars)- and lowest (14 cultivars)-Rb1 contents (*p* < 0.01) (Figure S1a). We identified a total of 1277 DETs (differential expression transcripts) (Table S1). These 1277 DETs had an average length of 2032 bp (Table S1), of which 771 were up-regulated, and 506 were down-regulated (Figure S1b). To further confirm the assembly of the DETs, we aligned them to the two ginseng draft genome assemblies and associated transcriptome assemblies (Databases IV and V) [5,6]. Of the 1277 DETs, all were aligned to the Korean ginseng genome assembly, while 88% were aligned to the Korean transcriptome assemblies [6]. All the 1277 DETs were also aligned to the Chinese ginseng genome assembly, and 81% were aligned to the Chinese ginseng transcriptome assemblies [5]. Therefore, these 1277 DETs were selected and designed as the Rb1 biosynthesis candidate gene DET-Is for further analysis.

Second, the 1277 Rb1 biosynthesis candidate gene DET-Is were further subjected to selection through Pearson’s correlation analysis between their expressions and Rb1 contents using all 42 representative ginseng cultivars. The expressions of 506 of the 1277 Rb1 biosynthesis candidate gene DET-Is were correlated with the variation of Rb1 contents among the 42 cultivars (*p* ≤ 0.05), of which 249 were correlated with the variations in the Rb1 contents among the 42 cultivars at a significance level of *p* ≤ 0.01 (Table S1). This result led to proceeding 506 of the Rb1 biosynthesis candidate gene DET-Is to Rb1 biosynthesis candidate gene DET-IIs.

**Figure 2:**
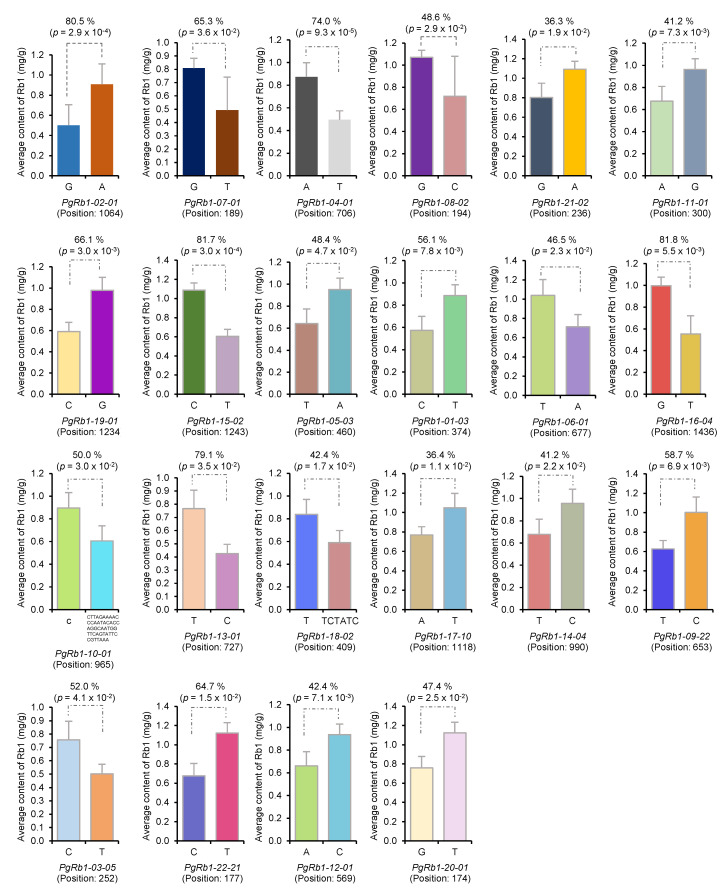
Biological impacts of the SNP/InDel mutations of the 22 DET-IIIs on Rb1 content in the four-year-old plant roots of 42 cultivars. Different colors indicate different SNP/InDel alleles. The mutation of each DET-III, e.g., *PgRb1-02-01*, is shown above the DET-III name, such as Nucleotide “G” relative “A”. The position of the mutation is indicated in parenthesis below the DET-III. The impact of a mutation is shown above each figure in percentage of Rb1 content difference between the two homozygous cultivar groups in the lower-Rb1 content cultivar group. For detail see Table S2.

Third, we conducted statistical analysis to determine whether the candidate gene DET-IIs had impacts on Rb1 contents if they mutated among the 42 cultivars. A total of 801 SNPs/InDels were identified in 111 of the 506 candidate gene DET-IIs, and no SNP/InDel was found in the remaining 395 candidate gene DET-IIs. Therefore, statistical analysis was conducted to determine the impacts of the 801 SNP/InDel mutations contained in the 111 candidate gene DET-IIs on Rb1 contents. Ninety-seven (12.1%) of the 801 genic SNPs/InDels, including 87 SNPs and 10 InDels, were found to have significant impacts on Rb1 contents (*p* ≤ 0.05), with an impact of each mutation on Rb1 content varying from 29.3% to 86.1% (Figure 2; Table S2). Of the 97 SNPs/InDels, 84 were within ORF (open-reading frame) and 13 were out of ORF. Fifty-nine of the 84 SNPs/InDels within ORF were non-synonymous (NS) SNPs, 15 were synonymous (S) SNPs, and 10 were ORF shift mutations. Of the 74 SNPs within ORF, 79.7% were “NS” mutations and 20.3% were “S” mutations. These 97 SNPs and InDels were contained in 22 of the 111 candidate gene DET-IIs, with an average of 4.4 SNPs/InDels per gene. These 22 candidate gene DET-IIs contained a total of 122 SNPs/InDels, 25 of which did not have impacts on Rb1 contents. Among these 97 SNPs/InDels, 34 had extremely significant impacts on Rb1 contents (*p* < 0.001). Given that this analysis method is independent of the identification of candidate gene DET-Is and DET-IIs that was based on gene transcript expression, the 506 candidate gene DET-IIs were identified from the 62,034 original transcripts, and that ginseng has a genome size of 3.3 Gb, the probability of identifying each of these 22 candidate gene DET-IIs that had a significant impact on Rb1 contents within a 1-Mb genomic region by chance is (506/62,034 × 1/3300) = 2.47 × 10^−6^, and the probability of simultaneously identifying all 22 associated candidate gene DET-IIs in the ginseng genome by chance is (506/62,034 × 10/3300)^22^ = 4.27 × 10^−102^, which is close to zero. Therefore, these 22 candidate gene DET-IIs that had significant impacts on Rb1 contents were selected as Rb1 biosynthesis candidate gene DET-IIIs for Rb1 biosynthesis.

**Figure 3:**
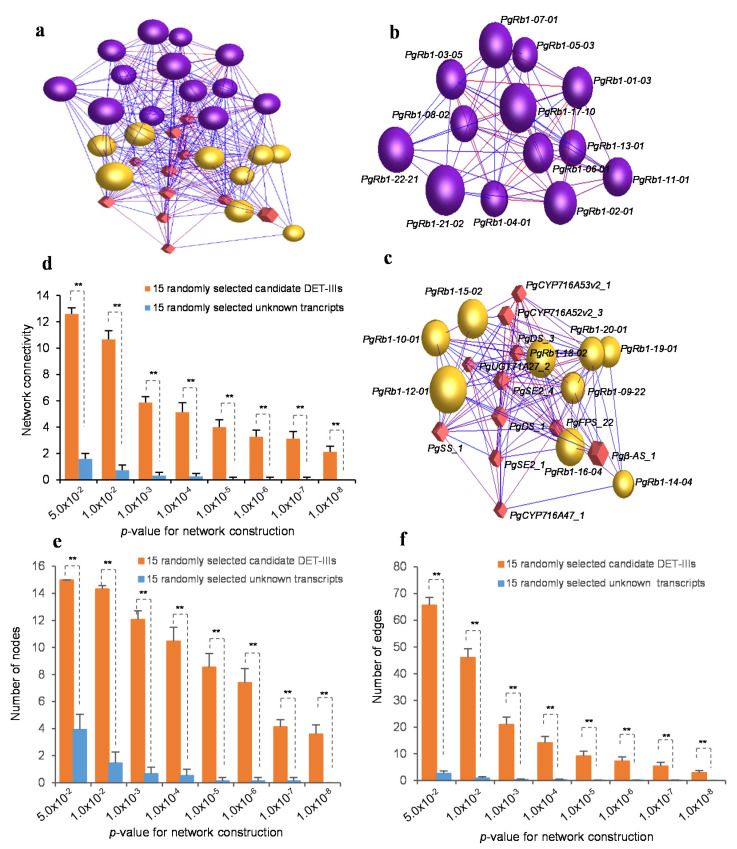
Co-expression network of 22 Rb1 candidate gene transcript DET-IIIs and 11 published Rb1 biosynthesis gene transcripts. (**a**) The co-expression network of the 33 gene transcripts constructed at a cutoff of *p* ≤ 0.05. The balls (nodes) represent Rb1 candidate gene transcript DET-IIIs, the diamonds (nodes) represent published Rb1 biosynthesis gene transcripts, and the lines (edges) between transcripts represent co-expressions or functional interactions. The network consists of all 22 Rb1 candidate gene transcript DET-IIIs and all 11 published Rb1 biosynthesis gene transcripts that form two clusters indicated by different colors. (**b**,**c**) The two clusters of the network, with each gene transcript being indicated. (**d**) The robustness of the Rb1 candidate gene transcript DET-III network relative to randomly selected unknown transcripts, indicated by network connectivity. The 15 candidate gene transcript DET-IIIs used for this experiment were randomly selected from the 22 candidate gene transcript DET-IIIs by bootstrap sampling with 20 replications. The 15 randomly selected unknown transcripts were selected from the ginseng expression database from which the 22 Rb1 candidate gene transcript DET-IIIs were identified, with 20 replications. (**e**) Tendency of the Rb1 candidate gene transcript DET-IIIs to form a co-expression network in number of nodes. (**f**) Tendency of the Rb1 candidate gene transcript DET-IIIs to form a co-expression network in number of edges. The connectivity of network, tendency of network formation in number of nodes, and tendency of network formation in number of edges were compared by *t*-test, with “**” for 2-tailed significant difference of *p* ≤ 0.01. Error bars, standard deviation.

Finally, we performed a co-expression network analysis of the 22 Rb1 biosynthesis candidate gene DET-IIIs with the 11 transcripts spliced from nine published genes controlling Rb1 biosynthesis (Table S3) to exclude the false positive genes, if any, from the 22 candidate gene DET-IIIs for Rb1 biosynthesis. All 22 candidate gene DET-IIIs formed a single co-expression network with all 11 published Rb1 biosynthesis gene transcripts (*p* < 0.05) consisting of two clusters (Figure 3a–c), even though the published Rb1 biosynthesis genes were cloned independently of the 22 candidate gene DET-IIIs and also of each other. The network was several-fold more likely to form a single co-expression network in terms of connectivity, number of nodes, and number of edges (Figure 3d–f). This result was the same as the findings discovered that the genes controlling a complex trait were several-fold more likely to form a single co-expression network [4]. Therefore, these 22 Rb1 biosynthesis candidate gene DET-IIIs were selected as candidate genes, designated *PgRb1* candidate genes. Each of the genes was coded with an Arabic number, and the transcript of the gene likely involved in Rb1 biosynthesis was coded by a suffix Arabic number, such as *PgRb1-01-03*, because different transcripts spliced from the same gene may have different biological functions [4,29].

### 2.3. Functional Validation of PgRb1 Candidate Genes for Rb1 Biosynthesis

**Figure 4:**
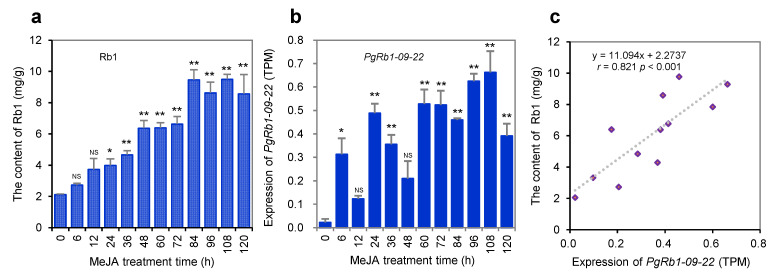
The 22 *PgRb1* candidate genes in the adventitious roots treated with MeJA for 6 h through 120 h, relative to the control roots not treated with MeJA (0 h). (**a**) Rb1 content variation in the adventitious roots treated with MeJA, relative to the control root. (**b**) The expression variation of one representative of the 22 *PgRb1* candidate genes, *PgRb1-09-22*, in the adventitious roots treated with MeJA, relative to the control root. (**c**) Correlation between the variation of the candidate gene expressions and the variation of Rb1 contents in the adventitious roots treated with and without MeJA. The “*” and “**” asterisks indicate that the difference of gene expression level between MeJA treated and control roots is significant at *p* ≤ 0.05 and 0.01, respectively. “NS” is for non-significant difference in Rb1 contents or expressions of the genes with the control root. For the expressions of the remaining 21 *PgRb1* candidate genes and their correlation with Rb1 contents, see Figures S2 and S3.

To confirm the functions of the 22 *PgRb1* candidate genes in Rb1 biosynthesis, we analyzed them by gene regulation with MeJA in adventitious roots because it has been widely used to validate the functions of candidate genes involved in ginsenoside biosynthesis in ginseng (see Materials and Methods). In comparison with the control treated without MeJA (sampled at the 0 h time point), the Rb1 contents in the adventitious roots were linearly increased from 0 h through 120 h as the treatment time lasted (Figure 4a). When the MeJA-treated adventitious roots were cultured for 24 h or longer, their Rb1 contents were significantly increased relative to those in the control roots (*p* ≤ 0.05 and 0.01). This result, therefore, confirmed the regulatory roles of MeJA in Rb1 biosynthesis and the robustness of the method for functional validation of candidate genes in ginsenoside biosynthesis. As Zhang et al. [29] showed that shot-gun RNA-seq is the method of choice to properly quantify gene and transcript expressions, we quantified the expressions of all gene transcripts expressed in the adventitious roots treated and not treated with MeJA by RNA-seq. Twenty-one of the 22 *PgRb1* candidate genes had significantly increased or decreased expressions at one or more of the 11 time points treated with MeJA, relative to the control sampled at 0 h (*p* ≤ 0.05 or 0.01), and only one, *PgRb1-20-01*, had an expression level not significantly different from the control (*p* > 0.05) (Figure 4b; Figure S2). This result validated that 21 (95.5%) of the 22 *PgRb1* candidate genes were involved in Rb1 biosynthesis; therefore, the 21 *PgRb1* candidate genes were considered the *PgRb1* genes controlling Rb1 biosynthesis.

Furthermore, we conducted a correlation analysis between the variation in the Rb1 contents and the expressions of the 22 *PgRb1* candidate genes to further confirm their roles in Rb1 biosynthesis. The result showed that the expression variations of eight of the 22 *PgRb1* candidate genes were correlated with Rb1 contents (*p* ≤ 0.05 or 0.01) (Figure 4c; Figure S3). These eight genes included *PgRb1-02-01*, *PgRb1-05-03*, *PgRb1-08-02*, *PgRb1-09-22*, *PgRb1-10-01*, *PgRb1-14-04*, *PgRb1-15-02*, and *PgRb1-21-02*. This result further confirmed that these genes controlled Rb1 biosynthesis.

### 2.4. Annotation and Gene Ontology (GO) Categorization of the PgRb1 Genes

We annotated and GO-categorized the 21 *PgRb1* genes identified in this study. Eighteen of the 21 *PgRb1* genes were annotated into different proteins, enzymes, and transcription factors, while three were not annotated (Table S4). GO analysis categorized the 18 *PgRb1* genes into 15 subcategories of all three primary categories, Cellular Component (CC) (6), Molecular Function (MF) (2), and Biological Process (BP) (7) (Figure S4). The *PgRb1* genes are especially involved in the CC’s cell part and cell, the MF’s catalytic activity and binding, and the BP’s metabolic process and cellular process. These results suggested that Rb1 biosynthesis is a complicated process involving diverse types of proteins, enzymes, and transcription factors.

### 2.5. Expression Mode of the 21 PgRb1 Genes with the 11 Published Rb1 Biosynthesis Genes

A total of 32 *PgRb1* genes, including the 21 *PgRb1* genes reported in this study, have thus far been cloned. To determine the relationships of our 21 *PgRb1* genes with the 11 published Rb1 biosynthesis genes in expression mode, we constructed and comparatively analyzed the expression heatmaps of the genes at different developmental stages of plant roots (Figure S5a), in different tissues of a four-year-old plant (Figure S5b), and in the four-year-old plant roots of different cultivars (Figure S5c). At the different developmental stages of plant roots, although some of the 21 *PgRb1* genes were clustered with some of the 11 published Rb1 biosynthesis genes, others formed clusters independent of the published Rb1 biosynthesis genes, suggesting that the *PgRb1* genes play unique roles in Rb1 biosynthesis. Notably, three of the newly cloned *PgRb1* genes, *PgRb1-17-10*, *PgRb1-05-03*, and *PgRb1-01-03*, had the same expression pattern from 5- through 25-year-old roots, indicating that they were co-regulated (Figure S5a). In the different tissues of a four-year-old plant (Figure S5b) and in the four-year-old plant roots of different cultivars (Figure S5c), the 21 *PgRb1* genes tended to be more clustered into groups even though a few of them were clustered with the published Rb1 biosynthesis genes, thus further confirming the unique roles of the *PgRb1* genes in Rb1 biosynthesis.

### 2.6. The Putative Pathways of the Rb1 Biosynthesis Genes in Rb1 Biosynthesis

**Figure 5:**
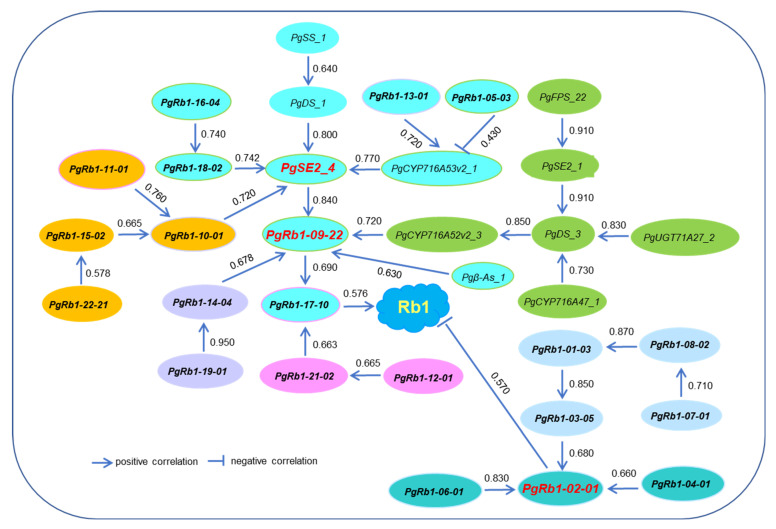
The putative pathway of 32 Rb1 biosynthesis genes in Rb1 biosynthesis. The pathway was determined by the correlations of their expressions in four-year-old plant roots of 42 cultivars at a cutoff *p*-value ≤ 0.01, assuming that the genes directly interacting with each other have higher expression correlations than those indirectly interacting. The correlation coefficients are shown between the genes. The genes highlighted with different colors indicate different interaction groups of the genes. The genes with bold font indicate the new *PgRb1* genes identified in this study and the remaining genes are the Rb1 biosynthesis genes previously cloned (Table S3). The genes in red bold font indicate the hub genes in the network. The arrow or “T” sign direction of the gene action is drawn based on its pathway toward Rb1.

Next, we deciphered how the 21 newly cloned *PgRb1* genes, along with the 11 published Rb1 biosynthesis genes, worked together to synthesize ginsenoside Rb1 based on the correlations of their expressions. Figure 5 shows the pathway of the 32 Rb1 biosynthesis genes in Rb1 biosynthesis. The 32 genes formed two sub-pathways (*r* = 0.430–0.910, *p* ≤ 0.01). One sub-pathway consisted of five interaction groups with a total of 25 of the 32 Rb1 genes, which positively contributed to Rb1 biosynthesis and had *PgSE2_4* and *PgRb1-09-22* as its hub genes. The other sub-pathway consisted of two interaction groups with a total of seven Rb1 genes, which negatively contributed to Rb1 biosynthesis and had *PgRb1-02-01* as its hub gene. The larger sub-pathway contained all 11 published Rb1 biosynthesis genes and 14 of the 21 newly cloned *PgRb1* genes. The smaller sub-pathway consisted of only seven of the 21 newly cloned *PgRb1* genes. This result agreed with the findings of their heatmap analysis that the newly cloned *PgRb1* genes played unique roles and interacted with the published Rb1 biosynthesis genes in Rb1 biosynthesis.

### 2.7. Co-Expression Network Variation of the Rb1 Biosynthesis Genes and its Impacts on Rb1 Biosynthesis

**Figure 6:**
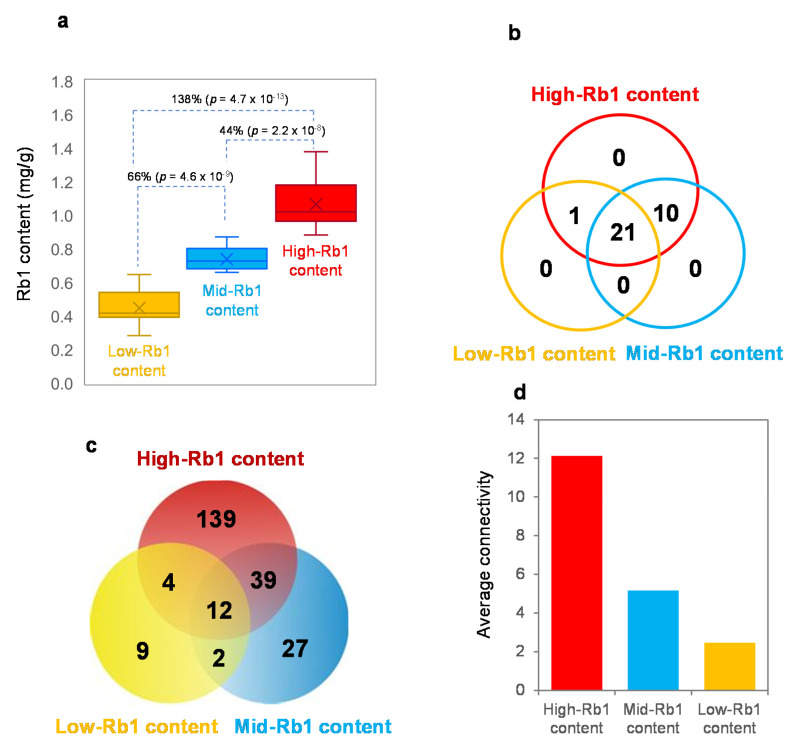
Impact of the Rb1 biosynthesis gene network variation on Rb1 biosynthesis. (**a**) Variation of Rb1 contents among cultivars. (**b**) Impact of variation of the gene nodes in the network on Rb1 biosynthesis. (**c**) Impact of variation of the gene interaction edges in the network on Rb1 biosynthesis. (**d**) Impact of variation of the network robustness, presented by average connectivity, on Rb1 biosynthesis.

The Rb1 content is a quantitative trait controlled by multiple genes. Zhang et al. [4] showed that the performance of a quantitative trait is a consequence of the interaction of the genes controlling the trait or gene epistasis. Therefore, we conducted a co-expression network analysis of the 21 *PgRb1* genes cloned in this study with the 11 published genes involved in Rb1 biosynthesis for cultivars with different Rb1 contents (Figure 6a). The 42 cultivars were grouped into three groups, including low-, mid-, and high-Rb1 content groups, with each group consisting of 14 cultivars, according to their Rb1 contents. The networks of the genes for each group were constructed and compared in the number of gene nodes (Figure 6b), the number of gene interaction edges (Figure 6c), and network robustness presented by connectivity (Figure 6d). All 32 genes involved in Rb1 biosynthesis formed a single co-expression network in the cultivar group with high-Rb1 contents. Nevertheless, the network of the genes for the cultivar group with mid-Rb1 contents consisted of only 31 of the 32 Rb1 biosynthesis genes analyzed, from which *Pgβ-AS_1* fell off. Consequently, the Rb1 contents decreased by 44%. Compared the mid-Rb1 content cultivar group with the low-Rb1 content cultivar group, the number of genes in the network was reduced from 31 to 22. Although *Pgβ-AS_1* was maintained in the gene network of the low-Rb1-content cultivar group, 10 additional genes of the 32 *PgRb1* genes fell off the network. This variation resulted in an additional reduction in Rb1 content by 66% (Figure 6a,b). The gene interaction edge variation in the network was also observed among the high-, mid-, and low-Rb1-content cultivar groups, varying from 194 to 80 and 27 edges. The edge variation in the networks was associated with the variation of Rb1 contents (Figure 6a,c). Finally, the connectivity of the networks appeared to vary with Rb1 contents. These results indicated that the Rb1 content was also a consequence of the interaction of the genes involved in Rb1 biosynthesis, and their network interaction greatly influenced Rb1 biosynthesis.

## 3. Discussion

The present study has developed and demonstrated a method for the rapid, accurate, efficient, and genome-wide identification of candidate genes controlling a biological trait or process using the ginsenoside Rb1 content in ginseng as a target trait. Genes are the central determinants of all traits and the core molecular basis of plant biology and genetic improvement. Therefore, extensive research has been conducted to develop methods that can identify candidate genes that likely control a biological trait or process. These methods include QTL mapping, eQTL mapping, GWAS, and gene DE analysis and have greatly contributed to the genome-wide identification of candidate genes for a biological trait or process. Nevertheless, as QTL mapping, eQTL mapping, and GWAS are linkage- or linkage disequilibrium (LD)-based, these methods cannot accurately identify candidate genes for a trait because a QTL interval or a genomic region often contains multiple genes, probably dozens to hundreds of genes. DE analysis is based on the expression of a gene that is essential for the gene to control a trait, but the candidate genes identified with the DE method often contain many false positive candidate genes due to the hypothesized relationship between gene expression and target trait. The analysis performed in this study is based on both the inherent relationship between gene transcript expression and trait performance and the impacts of the SNP/InDel mutations contained in the genes on the target trait and identifies candidate genes for a trait in a stepwise manner from four aspects of genes. These include transcript DE analysis between genotypes contrasting in the target trait, gene transcript expression–trait performance correlation analysis, and the impacts of gene mutations on the target trait, followed by gene transcript co-expression network analysis. Since a *p*-value = 0.001 for DE analysis, a *p*-value = 0.05 for gene transcript expression–trait phenotype variation correlation analysis, a *p*-value = 0.05 for impact analysis of gene mutation on the target trait, and a *p*-value = 0.05 for gene transcript co-expression network analysis were applied for the candidate gene identification for the trait, the collective *p*-value of these analyses of identifying a candidate gene by chance was 1.25 × 10^−7^, being close to zero. In addition, the expressions of individual transcripts were used for the DE, correlation, and co-expression network analyses because different transcripts spliced from a gene are shown to have different biological functions [4], which further increases the sensitivity and, thus, accuracy of candidate gene identification with these analyses. Furthermore, given that it is based on integrated and stepwise analysis of only transcriptomic and phenotypic data, the analysis is applicable to genome-wide and accurate identification of candidate genes controlling a biological trait or process in any species, including plants, animals, and microbes.

The utility and efficiency of the method developed in this study for genome-wide identification of candidate genes controlling a biological trait or process have been demonstrated by cloning 21 *PgRb1* genes involved in Rb1 biosynthesis. We have identified 22 candidate genes for Rb1 biosynthesis through the stepwise integrated analysis of transcriptomes with Rb1 contents in this study. The functions of 21 (95%) of them have been validated through MeJA regulation that has widely been used for functional validation of genes involved in ginsenoside biosynthesis in ginseng [8,13,14,15,16,17,18]. For instance, the functions of most of the key enzyme genes cloned thus far in ginsenoside biosynthesis were validated by gene regulation with MeJA, such as *PgSS* [21], *PgSE* [15], *PgMVD* [30], *PgFPS* [31], *PgDDS* [32], *PgUGT* [33], and *PgCYP450* [34]. Although the function of the one remaining candidate gene for Rb1 biosynthesis, *PgRb1-20-01*, has not been confirmed by the MeJA-mediated gene regulation method, it is only less than 5% out of the 22 candidate genes. Furthermore, we randomly selected one of the 21 *PgRb1* genes identified by MeJA regulation, *PgRb1-11-01*, and validated its function in Rb1 biosynthesis by the genetic transformation in another study (submitted). In addition, 96 genic SNPs/InDels were identified from these 21 *PgRb1* genes that had significant impacts on Rb1 content, providing an additional line of evidence on their function in Rb1 biosynthesis. These SNPs/InDels provide biomarkers that are the most desirable for the manipulation of Rb1 biosynthesis in ginseng and for enhanced ginseng breeding. Together, the results of all these experiments, including the MeJA regulation, genetic transformation, and mutation analysis, consistently conclude that the 21 *PgRb1* genes of the 22 candidate genes are involved in Rb1 biosynthesis.

Ginsenoside Rb1 biosynthesis has been shown to be a complicated process in which at least 32 genes, including the 21 *PgRb1* genes cloned in this study and 11 published Rb1 biosynthesis genes cloned in previous studies, are involved. First, the *PgRb1* genes encode a diverse class of proteins, enzymes, and transcription factors and are categorized into 15 secondary GO categories. The expressions of these *PgRb1* genes vary dramatically not only across tissues, developmental stages, and cultivars but also within a tissue, at a development stage, and in a cultivar. These results indicate that the genes involved in Rb1 biosynthesis have greatly diverged in nucleotide sequence, expression activity, and biochemical functionality, even though they are all involved in Rb1 biosynthesis. Second, the 21 *PgRb1* genes identified in this study with the 11 published Rb1 biosynthesis genes analyzed form two sub-pathways for Rb1 biosynthesis. One of the sub-pathways positively contributes to, and the other negatively contributes to, Rb1 biosynthesis. Third, network analysis reveals that the 32 Rb1 biosynthesis genes form a co-expression network several times stronger than that constructed from randomly selected unknown genes, suggesting that the process of Rb1 biosynthesis is completed by correlation of the genes controlling Rb1 biosynthesis in functionality. Moreover, this study reveals that the variation in the network has significantly influenced Rb1 biosynthesis. These findings are supported by previous studies of the molecular mechanisms underlying complex traits in ginseng [4,8,35], maize [4], and cotton [4]. Finally, the 32 genes do not seem to be all the genes involved in Rb1 biosynthesis because only the *PgRb1* genes that contain SNPs/InDels significantly impacting Rb1 biosynthesis have been identified in this study. The genes that do not contain SNPs/InDels but are involved in Rb1 biosynthesis cannot be identified through the analysis performed in this study. Therefore, additional research remains to identify the remaining genes involved in Rb1 biosynthesis and to comprehensively decipher the molecular mechanism underlying Rb1 biosynthesis.

## 4. Materials and Methods

### 4.1. Plant Materials

Four types of plant materials were used for this study. The first type of plant material was the four-year-old plant roots of 42 ginseng cultivars. Roots are the major organ where the ginsenosides, including Rb1, are synthesized and stored, thus the major products of ginseng. Although the older a ginseng plant, the higher the value of its root, farmers usually harvest roots at the four-year-old stage. These 42 cultivars were representative of the genetic diversity of ginseng in Jilin Province, China—the origin and the diversity center of *P. ginseng*. The Rb1 contents in the roots of the 42 cultivars were determined in our previous studies [35]. The second type of plant material was 14 tissues of a four-year-old ginseng plant (for details of the 14 tissues, see refs. [10,36]), and their Rb1 contents were previously determined [36]. The third type of plant material was the roots of 5-, 12-, 18-, and 25-year-old ginseng plants [10]. All these three types of plant materials were sampled from our ginseng field experimental station, Baishan, Jilin (41°56′38″ N 126°24′53″ E). The fourth type of plant material was the cultured adventitious roots of ginseng treated with methyl jasmonate (MeJA) (see below).

### 4.2. Database

Five databases, hereafter defined as I, II, III, IV, and V, were used in this study. Database I was derived from the four-year-old plant roots of the above 42 cultivars (NCBI/GEO 369 SRR13131364–SRR13131405). The database consists of the sequences of all gene transcripts expressed in the roots, the expressions of the transcripts and the genes, the single nucleotide polymorphism (SNP) and nucleotide insertion/deletion (InDel) mutations, and the contents of their ginsenosides, including Rb1 [8,35]. Database II was derived from the 14 tissues of a four-year-old ginseng plant. The database contains the sequences of all gene transcripts expressed in the 14 tissues (BioProject PRJNA302556) and the expressions of all transcripts spliced from the expressed genes, and the overall expressions of the genes (NCBI/GEO SRP066368) [10]. Database III was derived from the roots of the above 5-, 12-, 18-, and 25-year-old ginseng plants. It also consists of the sequences of all gene transcripts expressed in the roots and the expressions of all transcripts spliced from the expressed genes, and the overall expressions of the genes [10]. Database IV was the draft genome assembly of *P. ginseng* line IR826 [5]. Database V was the draft genome assembly of *P. ginseng* cv. ChP [6].

### 4.3. Genome-Wide Identification of Candidate Genes for Rb1 Biosynthesis

The candidate genes for Rb1 biosynthesis were identified by stepwise and integrated analysis of the database of the 42 cultivars (Database I) with four sequential steps. The analysis was designed based on two genetic bases: the central dogma of molecular biology (a gene controls a trait through its expression) and the impacts of gene mutations on the target trait (if a gene controls a trait, the mutation of the gene will influence its phenotype). Therefore, the two methods complement each other, and their combination allows much more accurate and efficient identification of candidate genes than either method alone for a trait or biological process.

Step 1: Transcript differential expression analysis between cultivars with highest- and lowest-Rb1 contents in roots. This is the first step of the integrated analysis of transcriptomes with Rb1 contents for genome-wide identification of candidate genes for Rb1 biosynthesis. Zhang et al. [29] and Han et al. [36] showed that most genes are subjected to RNA alternative splicing, forming multiple transcripts from a single gene while they express in a tissue at a developmental stage in a genotype. Zhang et al. [4] discovered that different transcripts spliced from a gene have different biological functions. Therefore, the expressions of transcripts were used for this analysis so that the sensitivity of identifying the candidate genes controlling a biological trait could be substantially increased. The 42 cultivars were sorted in ascending according to the Rb1 contents of their roots. The 14 highest- and 14 lowest-Rb1 content cultivars were selected and subjected to Student’s *t*-test. Statistical analysis showed the Rb1 contents between the 14 highest and 14 lowest cultivars were significantly different (*p* < 0.01). Therefore, we sampled cultivars from each of the two groups, respectively, five times with a bootstrap replacement sampling method in which some of the 14 cultivars in each group might be sampled more than once while others might not be sampled at all. Consequently, we achieved five bootstrap replications, with each consisting of 14 cultivar samples. The expressions of the transcripts in the roots of the 14 highest- and 14 lowest-Rb1 content cultivars were extracted from Database I. The mean expression of each transcript in the 14 cultivar samples was calculated and used as a bootstrap replicate. Therefore, we had five bootstrap replicates for each of the highest- and lowest-Rb1 content cultivar groups for transcript differential expression analysis. The analysis was conducted with DESeq2 v1.32.0 [37]. The transcript with differential expression by log_2_ (foldchange) ≥ 2 and adjusted *p*-value ≤ 0.001 between the two groups was counted as a candidate gene I for Rb1 biosynthesis, defined as candidate gene DET-I.

Step 2: Correlation analysis between the expressions of the candidate gene DET-Is and Rb1 contents. Zhang et al. [4] showed that the expressions of nearly 100% of the gene transcripts controlling a quantitative trait were correlated with the phenotype of the trait, no matter what genetic resources they were cloned from; thus, correlation analysis between gene transcript expression and phenotype variation of a trait provides a line of evidence on the candidate genes controlling the trait. All 42 cultivars selected above were used for this experiment. The expression of each DET-I in the 42 cultivars was subjected to correlation analysis with their Rb1 contents using the SPSS package (IBM SPSS Statistics 23). The candidate gene DET-I whose expression was significantly correlated with the Rb1 content among the 42 cultivars was selected as candidate gene II for Rb1 biosynthesis, defined as candidate gene DET-II.

Step 3: Biological impacts of the SNPs/InDels of the DET-IIs on Rb1 contents. We hypothesized that if a gene controls a trait, the mutation of the gene will influence its phenotype. All 42 cultivars were used for this experiment. The genic SNPs/InDels of the candidate gene DET-IIs were extracted from the genic SNP/InDel dataset of Database I, which were called by the SAMtools software using the transcript sequences of Database II (developed from 14 tissues of a four-year-old cv Damaya plant) [38,39] as the reference. The impact of each DET-II genic SNP/InDel on Rb1 content was determined statistically. Briefly, the 42 cultivars were grouped using the genotypes of the SNP under study. Since every SNP is bi-allelic, the 42 cultivars were grouped into two groups if no heterozygote existed among the 42 cultivars or three groups if heterozygotes were identified among the 42 cultivars. If the 42 cultivars were grouped into two groups, Student’s *t*-test was used to determine whether the SNP or InDel under study had an impact on Rb1 contents. If the 42 cultivars were grouped into three groups, one-way analysis of variance (ANOVA) was used to determine whether the SNP or InDel under study had an impact on Rb1 contents. The candidate gene DET-II with an SNP or InDel that had a significant impact on Rb1 content (*p* ≤ 0.05) was selected and defined as candidate gene DET-III for Rb1 biosynthesis. Moreover, the degree of impact of the SNP or InDel mutation on Rb1 content was estimated using the following formula:

Y_i_ = (G_ih_ − G_il_)/G_il_ × 100

where Y_i_ is the biological impact of mutation i on the Rb1 content, G_ih_ is the mean Rb1 content of the homozygous group with higher Rb1 content, and G_il_ is the mean Rb1 content of the homozygous group with lower Rb1 content. Finally, the type of the SNP or InDel, such as synonymous SNP (nucleotide substitution) and non-synonymous SNP, was classified by the ORF (open-reading frame) Finder.

Step 4: Co-expressions of the candidate gene DET-IIIs with previously published Rb1 biosynthesis genes. Zhang et al. [4] showed that the gene transcripts controlling a quantitative trait were several times more likely to form a co-expression network than randomly selected unknown gene transcripts. Therefore, we further conducted the co-expression network analysis of the candidate gene DET-IIIs with the previously published Rb1 biosynthesis genes (Table S3). Zhao et al. [35] previously conducted a correlation analysis between expressions of 16 transcripts spliced from 10 published ginsenoside biosynthesis genes and Rb1 contents. They showed that 11 of the transcripts spliced from nine of the published ginsenoside biosynthesis genes were significantly correlated in expressions with Rb1 contents. Therefore, the expressions of the candidate gene DET-IIIs and the published Rb1 biosynthesis gene transcripts were both extracted from the transcript expression dataset of Database I that were counted by RNA-seq, followed by transcript quantification with the RSEM software [40]. The co-expression network was constructed using the Biolayout *Express*^3D^ (Version 3.3) [41] and characterized as previously described [4,8,35]. The candidate gene DET-IIIs that formed a strong co-expression network with the published Rb1 biosynthesis gene transcripts were selected and defined as *PgRb1* candidate genes for Rb1 biosynthesis.

### 4.4. Functional Validation of PgRb1 Candidate Genes for Rb1 Biosynthesis

Because roots are the major organ in which ginsenosides are synthesized and stored, gene regulation with MeJA in cultured adventitious roots has widely been used to functionally validate genes that are involved in ginsenoside biosynthesis in ginseng [8,13,14,15,16,17,18]. The functions of most of the key enzyme genes cloned thus far in ginsenoside biosynthesis were validated by this method, including *PgMVD* [30], *PgFPS* [31], *PgDDS* [32], *PgSS* [21], *PgSE* [15], *PgUGT* [33], and *PgCYP450* [34]. Therefore, we validated the roles of the *PgRb1* candidate genes in Rb1 biosynthesis using this method. Ginseng seeds were used as the sources of plant materials for adventitious root induction. The coats of the ginseng seeds were removed, washed, and sterilized. The embryos of the seeds were carefully excised and then cultured on 1/2 MS (Murashige and Skoog) agar basal medium containing 1.0 mg/L gibberellins at 22–25 °C under 14 h light /10 h dark for one week to generate sterile seedlings. The healthy sterile seedlings were selected, and their leaflet pedicels were cut into approximately 0.5 cm segments under sterile conditions and cultured on the MS agar medium containing 2 mg/L 2,4-D (2,4-dichlorophenoxyacetic acid) and 0.2 mg/L 6-BA (6-benzylaminopurine) at 22–25 °C in the dark for 4–5 weeks to induce calli. The vigorous calli were selected, inoculated onto B5 (Gamborg’s B-5) agar basal medium containing 3.0 mg/L IBA (indole-3-butyric acid), and cultured at 22–25 °C in the dark to induce adventitious roots. Vigorous adventitious roots were individually selected and cultured in a B5 liquid medium at 22–25 °C, 110 RPM in the dark for 30 days to reproduce adventitious roots.

The 30-old-day vigorous adventitious roots were selected, inoculated into 250 mL fresh B5 liquid medium with an amount of 1.0 g adventitious roots per 250 mL medium, and cultured at 22–25 °C, 110 RPM in the dark for 25 days. Since the experiment contained 12 treatment time points with MeJA, with each time point having three biological replicates, a total of 36 flasks containing 250 mL B5 liquid medium were inoculated and cultured. On the 25th day of the culture, MeJA was added to 33 of the 36 adventitious root culture flasks, respectively, at a final concentration of 200 µM MeJA. The adventitious roots were then harvested at 0 h (the culture with no MeJA added), 6 h, 12 h, 24 h, 36 h, 48 h, 60 h, 72 h, 84 h, 96 h, 108 h, and 120 h. One gram of the adventitious roots harvested from each of the three replicates at each time point was immediately frozen in liquid nitrogen and stored at −80 °C for RNA analysis. The remaining adventitious roots were dried and stored at 4 °C for ginsenoside analysis.

Ginsenosides were extracted from all three biological replicates of the dried adventitious roots sampled at each time point by the Soxhlet extraction method, as described in previous studies [42]. One gram of each dried root replicate sample was used for ginsenoside extraction. Mono-ginsenosides were separated using the Waters Alliance HPLC (high-performance liquid chromatography) with an e2695 Separation Module. The contents of individual mono-ginsenosides were determined using the Waters 2489 Ultraviolet Spectrophotometric Detector (Waters, Milford, MA, USA), according to Li et al. [42]. The contents of Rb1 were extracted from the measurement results of the mono-ginsenosides. To validate the effects of the MeJA treatment on Rb1 biosynthesis, the contents of Rb1 that had three biological replicates for each of 6 h through 120 h time points were compared with the contents of Rb1 for the 0 h time point (non-MeJA treatment control that also had three biological replicates) by Student’s *t*-test.

The total RNAs were isolated from all three biological replicates of the frozen adventitious roots sampled at each time point using the TRIpure Reagent Total RNA Extraction Reagent (Bioteke, Beijing, China). RNA-seq libraries were constructed as described by Zhang et al. [29]. The libraries were qualified, quantified, multiplexed, and sequenced using the HiSeq X Ten (Illumina, Inc., San Diego, USA), with 150 PE (paired-end) and >30 million clean reads per sample. The sequence reads were quality filtered and trimmed as described by Zhang et al. [29] before further analysis. Individual gene transcripts were assembled from the clean reads using the Trinity v2.14.0 software [43] with the transcriptome assembly of Database II as the reference. The expressions of individual transcripts and the overall expressions of genes were quantified using the RSEM v1.3.3 software [40]. The expressions of the transcripts in transcripts per million (TPM) were used for further analysis. The expressions of the *PgRb1* candidate gene transcripts were extracted from each biological replicate of the adventitious roots sampled at each time point. The effects of the MeJA treatment on the expressions of the *PgRb1* candidate genes were confirmed by comparing the expressions of the genes in the MeJA-treated samples at each time point with those in the control samples at the 0 h time point by Student’s *t*-test.

To further confirm the roles of the *PgRb1* candidate genes in Rb1 biosynthesis, the expressions of their individual transcripts at each time point, including the 0 h time point, were subjected to Pearson’s correlation analysis with the Rb1 contents at each time point. The expressions of the individual transcripts spliced from the *PgRb1* candidate genes were used for the correlation analysis because different transcripts spliced from a single gene may have different functions [4]. If a transcript expression of a *PgRb1* candidate gene was correlated with the Rb1 contents at a two-tailed significance level of *p* ≤ 0.05, the gene spliced into the transcript was considered as the gene involved in Rb1 biosynthesis.

### 4.5. Annotation and Gene Ontology (GO) Categorization of the 21 PgRb1 Genes

The *PgRb1* genes were annotated and categorized in GO using the Blast2go 5.2 software [44].

### 4.6. Expression Mode of the 21 PgRb1 Genes with the 11 Published Rb1 Biosynthesis Genes

To have a first insight into the functional relationships of the Rb1 biosynthesis genes in Rb1 biosynthesis, we constructed their expression heatmaps in different tissues of a four-year-old plant, at different developmental stages in plant roots, and in the four-year-old plant roots among cultivars. The expressions of the *PgRb1* genes in different tissues, at different developmental stages, and across cultivars were extracted from the expression datasets of Databases II, III, and I, respectively. The expression heatmaps in different tissues, at different developmental stages, and across cultivars were constructed using the R programming language and software, according to Wang et al. [10].

### 4.7. The Putative Pathways of the Rb1 Biosynthesis Genes in Rb1 Biosynthesis

Moreover, we inferred the pathway of the Rb1 biosynthesis genes in Rb1 biosynthesis. Pearson’s correlation coefficients of the expressions between the genes and Rb1 content were calculated using the SPSS package (IBM SPSS Statistics 23). We hypothesized that the genes that directly interacted in Rb1 biosynthesis tended to have higher correlation coefficients of expressions than the genes that indirectly interacted in Rb1 biosynthesis in a group of genes that interacted. Therefore, the interaction groups of the genes were constructed first and then connected based on their expression correlation coefficients. The gene that is in the central position of the pathway, assuming that it was playing a central role, was defined as a hub gene. The action direction of a gene in a pathway was assigned toward ginsenoside Rb1.

### 4.8. Co-Expression Network Variation of the Rb1 Biosynthesis Genes and its Impacts on Rb1 Biosynthesis

Finally, we examined their co-expression network with the previously published Rb1 biosynthesis genes and the impact of the network variation on Rb1 contents to determine the relationship of the *PgRb1* genes in Rb1 biosynthesis and the impacts of their relationship variation on Rb1 biosynthesis. The 42 cultivars were grouped into three groups that differed in Rb1 content (*p* ≤ 0.01), with low-, mid-, and high-Rb1 contents, and each group containing 14 cultivars. The co-expression networks of the *PgRb1* genes were constructed for each group of cultivars separately, using the BioLayout *Express*^3D^ software [41] as described above. The resultant co-expression networks of the genes were compared among the three groups of cultivars in major characteristics of the networks, including the number of gene nodes, the number of gene interactions or co-expression edges, and network robustness presented by connectivity.

## Data Availability

The data used for this study are available at the Sequence Read Archive (SRA) of the National Center for Biotechnology Information (NCBI), BioProject PRJNA302556; and at Gene Expression Omnibus (GEO) of NCBI, SRP066368 and SRR13131364-SRR13131405. The plant materials are available through corresponding authors upon request.

## Supplementary Materials

The following supporting information can be downloaded at: https://www.mdpi.com/article/10.3390/ijms232214016/s1.
