# Supplementary material for: Transcriptome and Phenotype Integrated Analysis Identifies Genes Controlling Ginsenoside Rb1 Biosynthesis and Reveals Their Interactions in the Process in Panax ginseng
Source: Int J Mol Sci. 2022 Nov 13;23(22):14016. doi: 10.3390/ijms232214016 (PMC9698431; doi:10.3390/ijms232214016)
Supplement: Supplementary file 1 [file ijms-23-14016-s001.zip › FigS1_Jiang et al._DE.pptx]

## Slide 1
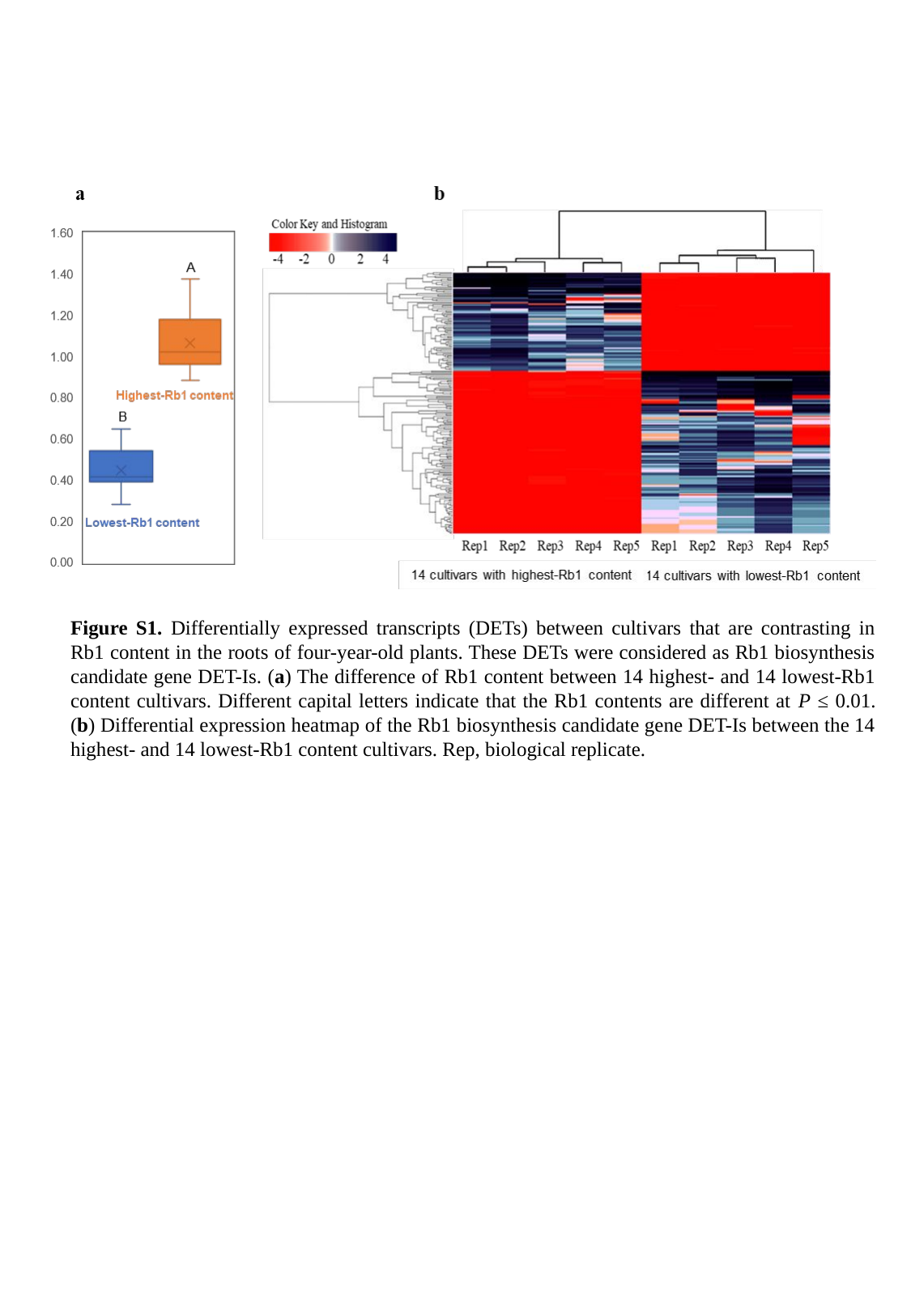

Figure S1. Differentially expressed transcripts (DETs) between cultivars that are contrasting in Rb1 content in the roots of four-year-old plants. These DETs were considered as Rb1 biosynthesis candidate gene DET-Is. (a) The difference of Rb1 content between 14 highest- and 14 lowest-Rb1 content cultivars. Different capital letters indicate that the Rb1 contents are different at P ≤ 0.01. (b) Differential expression heatmap of the Rb1 biosynthesis candidate gene DET-Is between the 14 highest- and 14 lowest-Rb1 content cultivars. Rep, biological replicate.
