# Supplementary material for: Transcriptome and Phenotype Integrated Analysis Identifies Genes Controlling Ginsenoside Rb1 Biosynthesis and Reveals Their Interactions in the Process in Panax ginseng
Source: Int J Mol Sci. 2022 Nov 13;23(22):14016. doi: 10.3390/ijms232214016 (PMC9698431; doi:10.3390/ijms232214016)
Supplement: Supplementary file 1 [file ijms-23-14016-s001.zip › FigS2_Jiang et al._22 expressions-MeJA.pptx]

## Slide 1
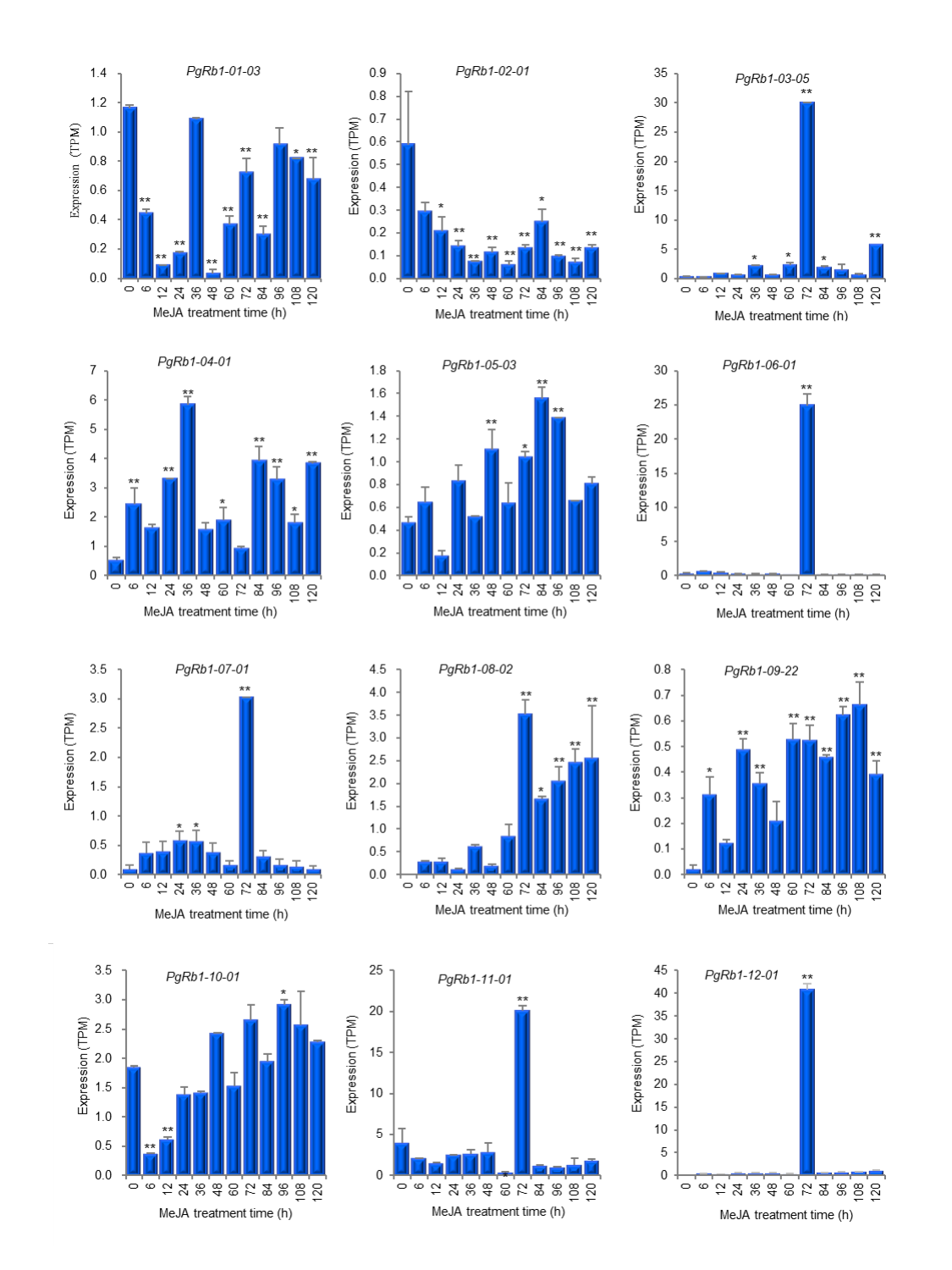

## Slide 2
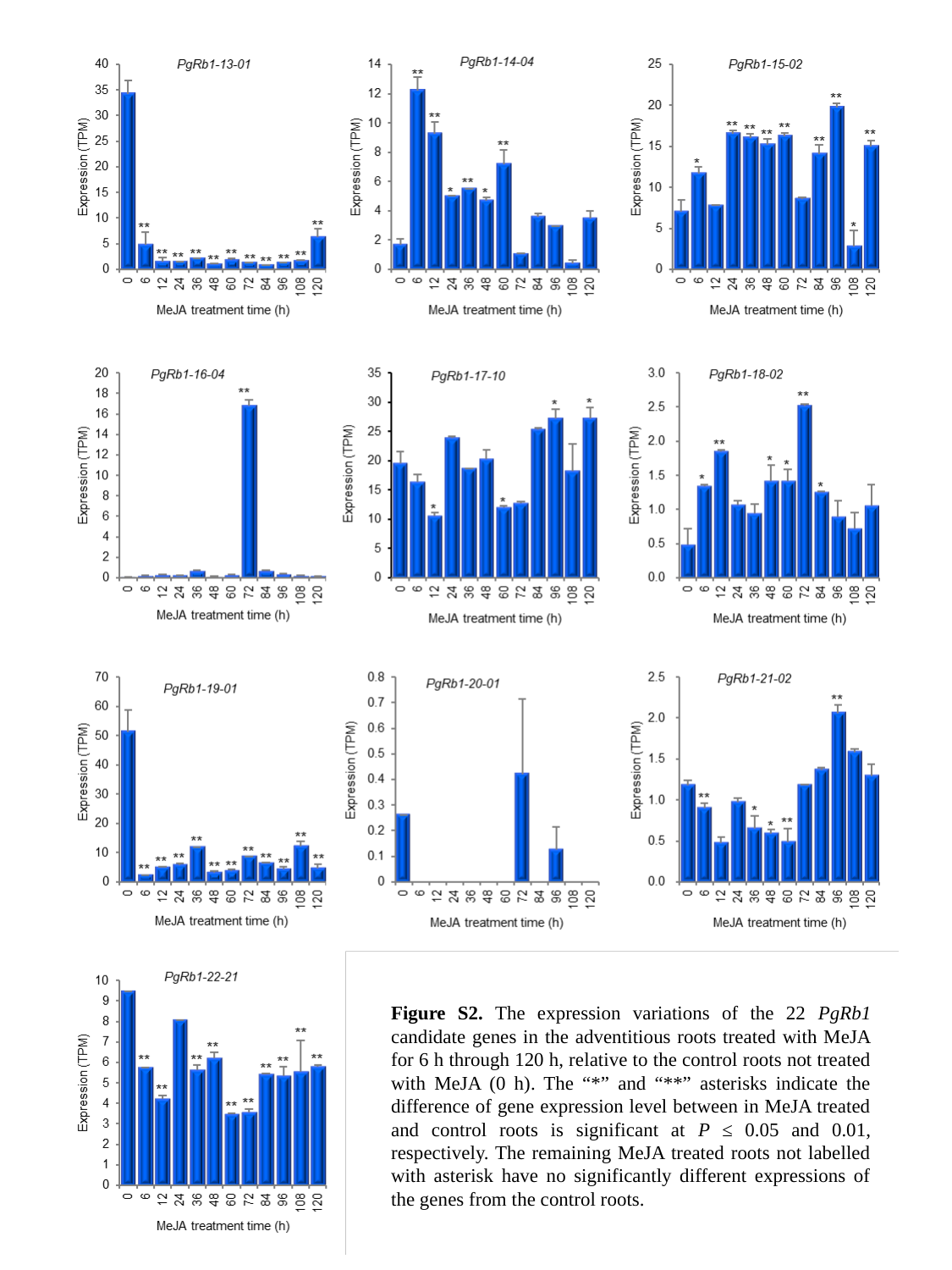

Figure S2. The expression variations of the 22 PgRb1 candidate genes in the adventitious roots treated with MeJA for 6 h through 120 h, relative to the control roots not treated with MeJA (0 h). The “*” and “**” asterisks indicate the difference of gene expression level between in MeJA treated and control roots is significant at P ≤ 0.05 and 0.01, respectively. The remaining MeJA treated roots not labelled with asterisk have no significantly different expressions of the genes from the control roots.
