# Supplementary material for: Transcriptome and Phenotype Integrated Analysis Identifies Genes Controlling Ginsenoside Rb1 Biosynthesis and Reveals Their Interactions in the Process in Panax ginseng
Source: Int J Mol Sci. 2022 Nov 13;23(22):14016. doi: 10.3390/ijms232214016 (PMC9698431; doi:10.3390/ijms232214016)
Supplement: Supplementary file 1 [file ijms-23-14016-s001.zip › FigS3_Jiang et al._correlation_MeJA.pptx]

## Slide 1
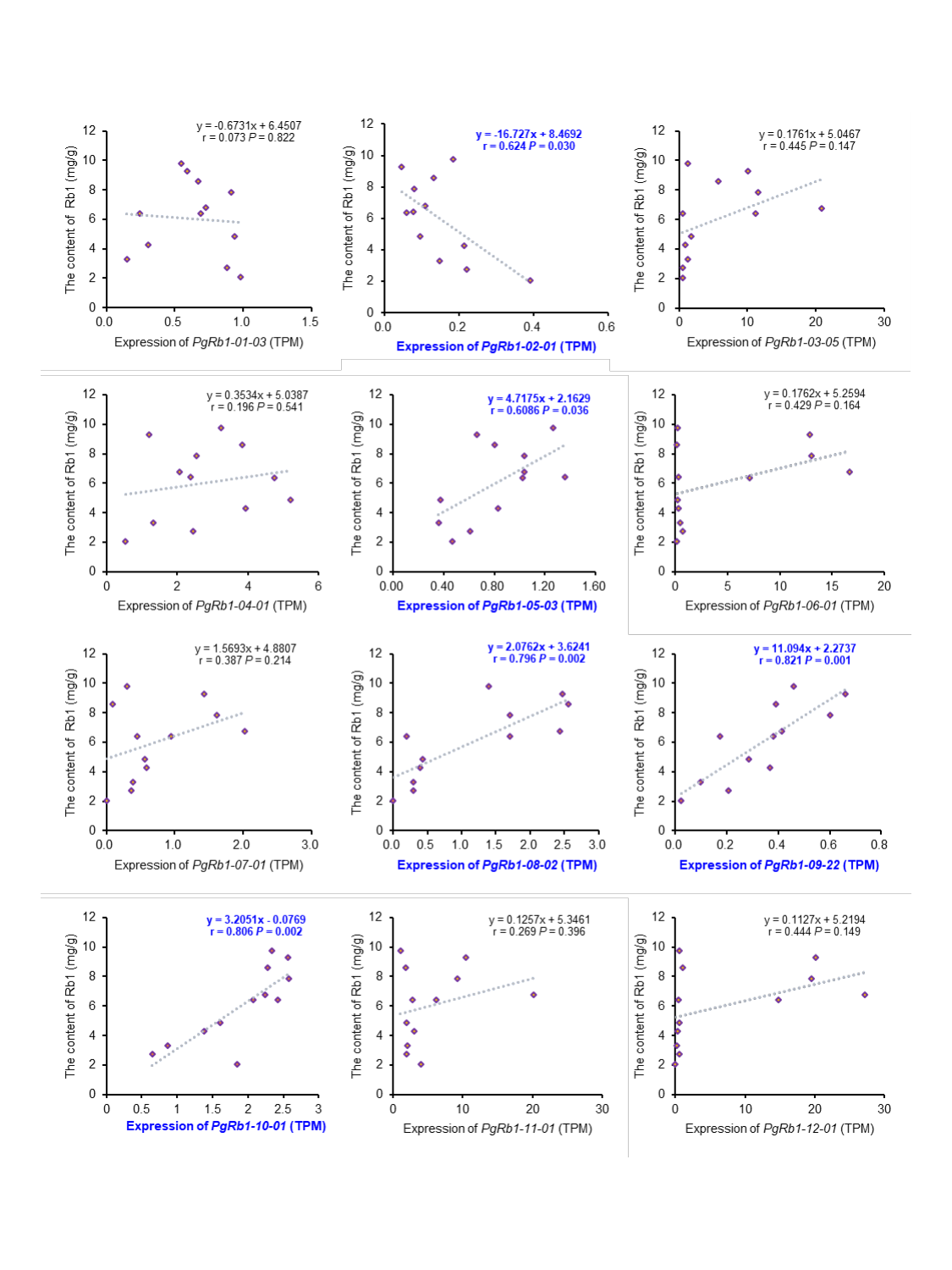

## Slide 2
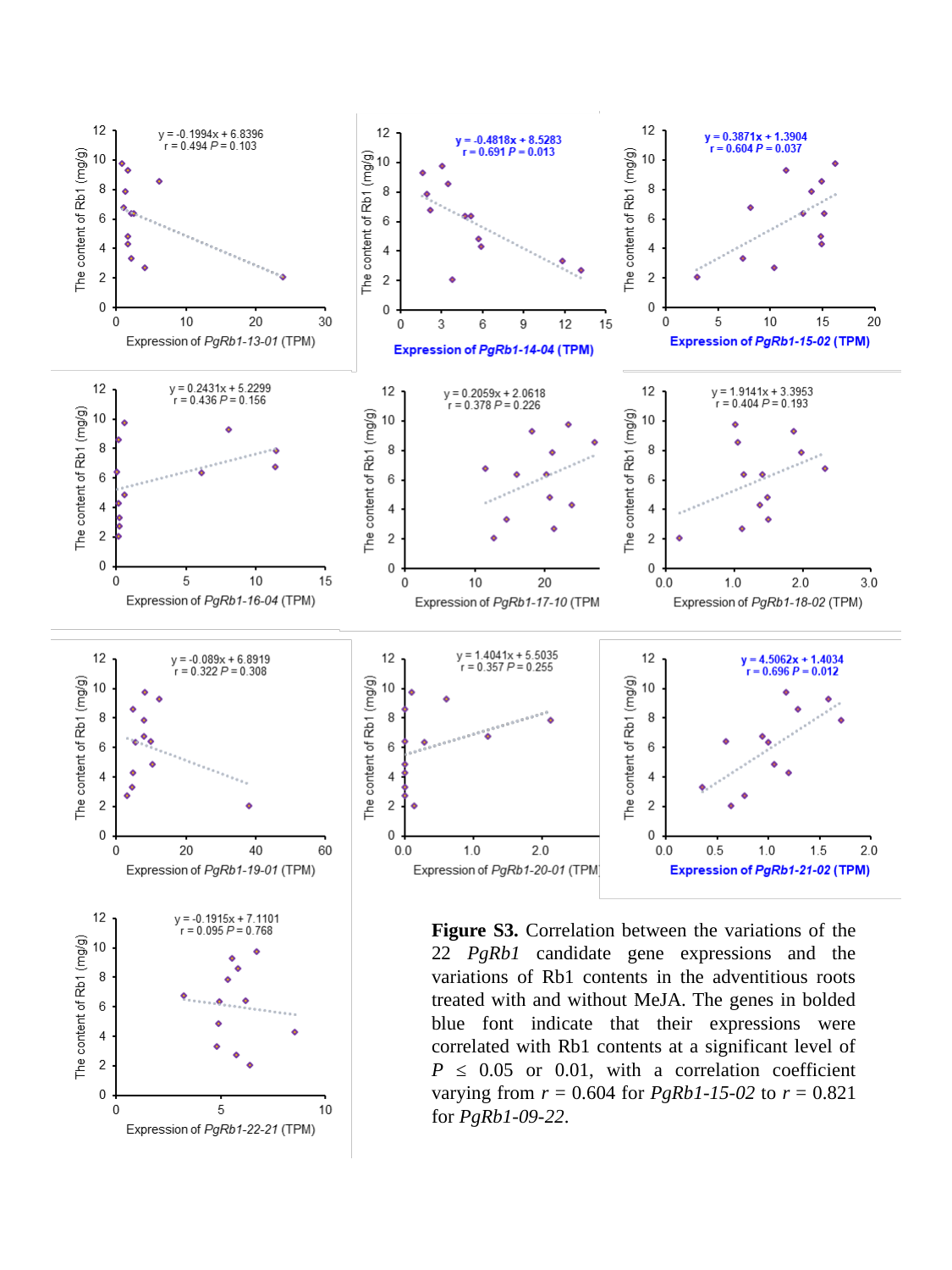

Figure S3. Correlation between the variations of the 22 PgRb1 candidate gene expressions and the variations of Rb1 contents in the adventitious roots treated with and without MeJA. The genes in bolded blue font indicate that their expressions were correlated with Rb1 contents at a significant level of P ≤ 0.05 or 0.01, with a correlation coefficient varying from r = 0.604 for PgRb1-15-02 to r = 0.821 for PgRb1-09-22.
