# Supplementary material for: Transcriptome and Phenotype Integrated Analysis Identifies Genes Controlling Ginsenoside Rb1 Biosynthesis and Reveals Their Interactions in the Process in Panax ginseng
Source: Int J Mol Sci. 2022 Nov 13;23(22):14016. doi: 10.3390/ijms232214016 (PMC9698431; doi:10.3390/ijms232214016)
Supplement: Supplementary file 1 [file ijms-23-14016-s001.zip › FigS4_Jiang et al._GO.pptx]

## Slide 1
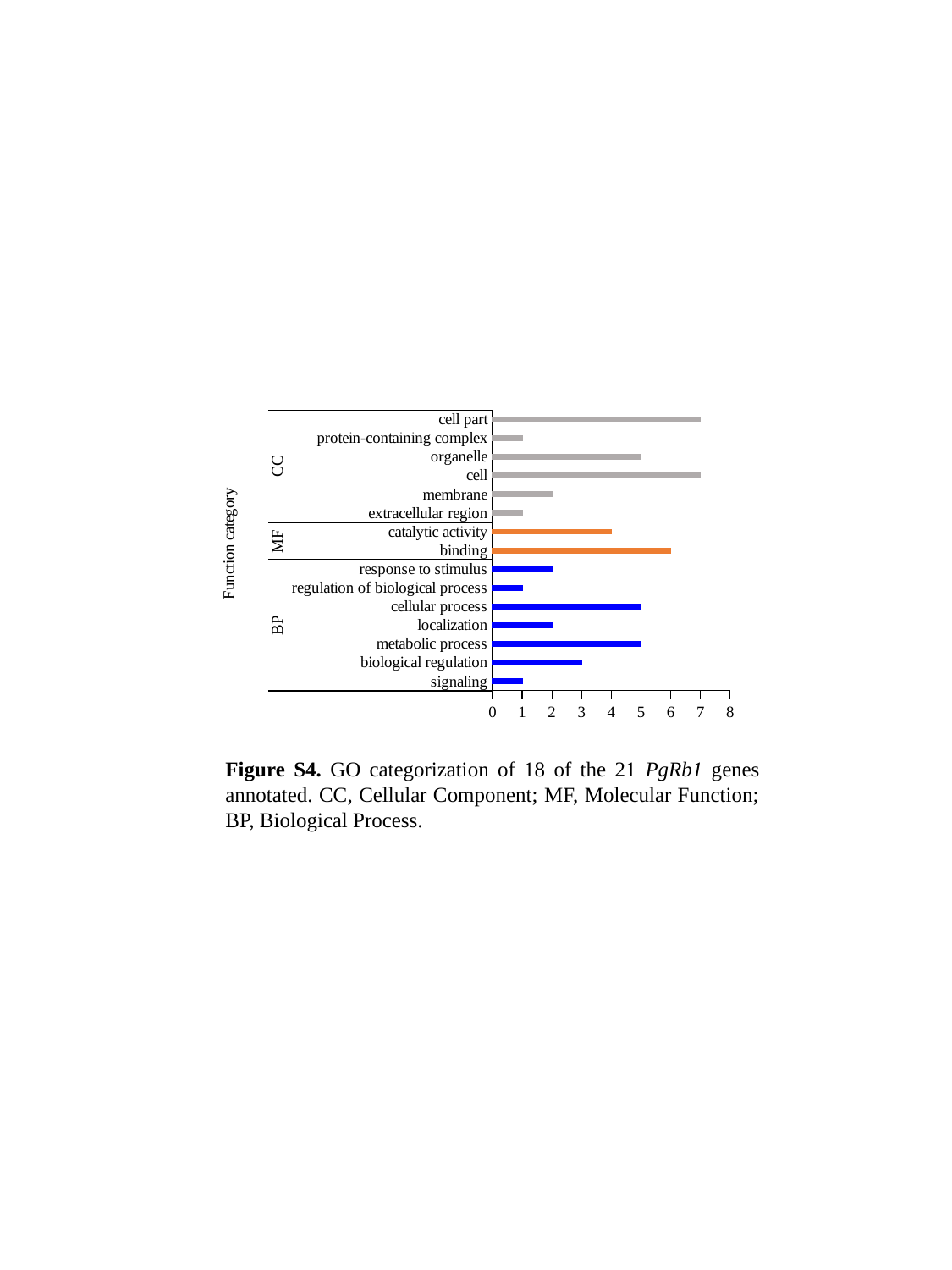

### Chart
| Category | |
|---|---|
| signaling | 1.0 |
| biological regulation | 3.0 |
| metabolic process | 5.0 |
| localization | 2.0 |
| cellular process | 5.0 |
| regulation of biological process | 1.0 |
| response to stimulus | 2.0 |
| binding | 6.0 |
| catalytic activity | 4.0 |
| extracellular region | 1.0 |
| membrane | 2.0 |
| cell | 7.0 |
| organelle | 5.0 |
| protein-containing complex | 1.0 |
| cell part | 7.0 |Figure S4. GO categorization of 18 of the 21 PgRb1 genes annotated. CC, Cellular Component; MF, Molecular Function; BP, Biological Process.
