# Supplementary material for: Transcriptome and Phenotype Integrated Analysis Identifies Genes Controlling Ginsenoside Rb1 Biosynthesis and Reveals Their Interactions in the Process in Panax ginseng
Source: Int J Mol Sci. 2022 Nov 13;23(22):14016. doi: 10.3390/ijms232214016 (PMC9698431; doi:10.3390/ijms232214016)
Supplement: Supplementary file 1 [file ijms-23-14016-s001.zip › FigS5_Jiang et al._heatmap.pptx]

## Slide 1
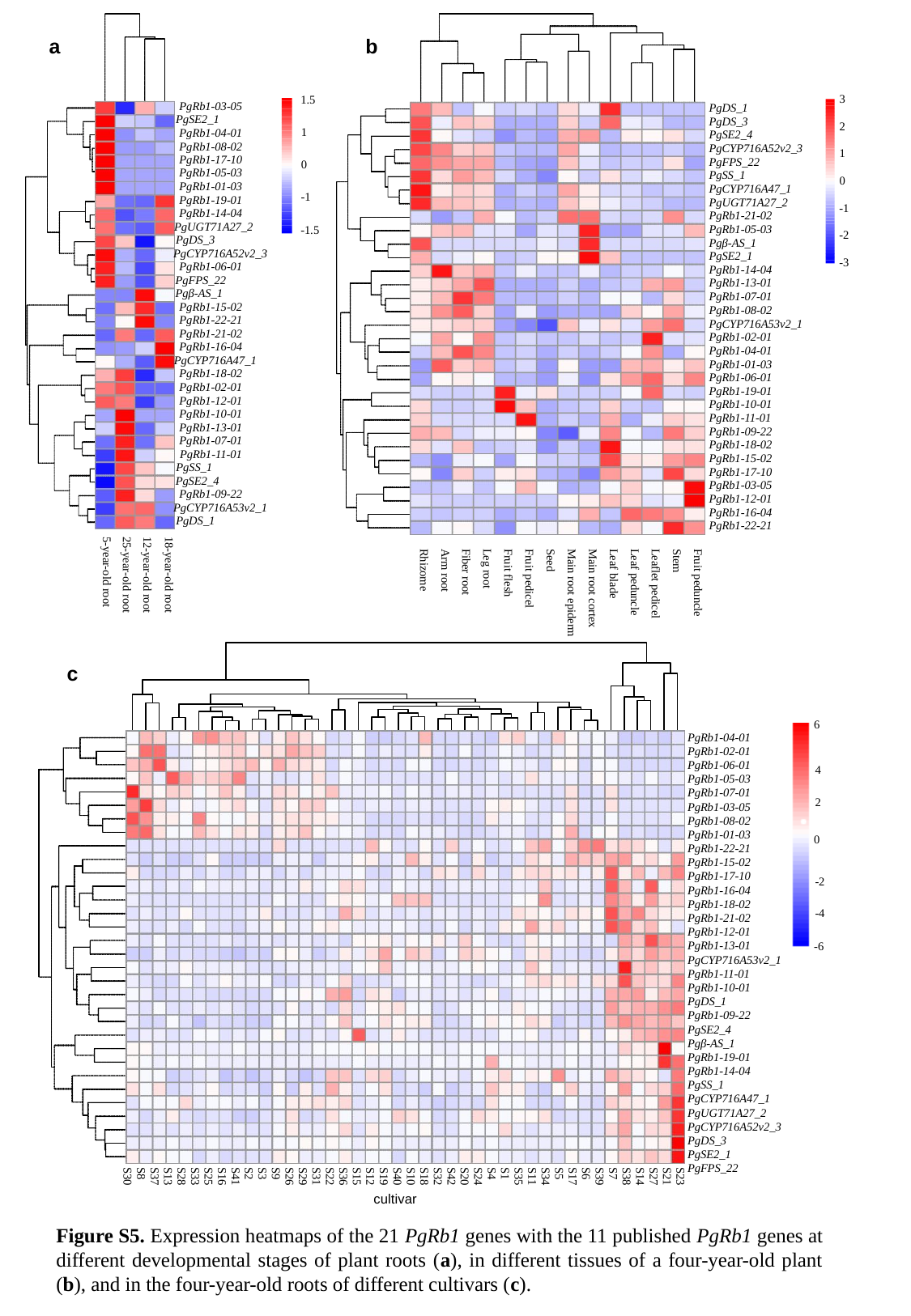

1.5
1
0
-1
-1.5
PgRb1-03-05
PgSE2_1
PgRb1-04-01
PgRb1-08-02
PgRb1-17-10
PgRb1-05-03
PgRb1-01-03
PgRb1-19-01
PgRb1-14-04
PgUGT71A27_2
PgDS_3
PgCYP716A52v2_3
PgRb1-06-01
PgFPS_22
Pgβ-AS_1
PgRb1-15-02
PgRb1-22-21
PgRb1-21-02
PgRb1-16-04
PgCYP716A47_1
PgRb1-18-02
PgRb1-02-01
PgRb1-12-01
PgRb1-10-01
PgRb1-13-01
PgRb1-07-01
PgRb1-11-01
PgSS_1
PgSE2_4
PgRb1-09-22
PgCYP716A53v2_1
PgDS_1
5-year-old root
25-year-old root
12-year-old root
18-year-old root
3
2
1
0
-1
-2
-3
PgDS_1
PgDS_3
PgSE2_4
PgCYP716A52v2_3
PgFPS_22
PgSS_1
PgCYP716A47_1
PgUGT71A27_2
PgRb1-21-02
PgRb1-05-03
Pgβ-AS_1
PgSE2_1
PgRb1-14-04
PgRb1-13-01
PgRb1-07-01
PgRb1-08-02
PgCYP716A53v2_1
PgRb1-02-01
PgRb1-04-01
PgRb1-01-03
PgRb1-06-01
PgRb1-19-01
PgRb1-10-01
PgRb1-11-01
PgRb1-09-22
PgRb1-18-02
PgRb1-15-02
PgRb1-17-10
PgRb1-03-05
PgRb1-12-01
PgRb1-16-04
PgRb1-22-21
Rhizome
Arm root
Fiber root
Leg root
Fruit flesh
Fruit pedicel
Seed
Main root epiderm
Main root cortex
Leaf blade
Leaf peduncle
Leaflet pedicel
Stem
Fruit peduncle
a
b
6
4
2
0
-2
-4
-6
PgRb1-04-01 PgRb1-02-01 PgRb1-06-01 PgRb1-05-03 PgRb1-07-01 PgRb1-03-05 PgRb1-08-02 PgRb1-01-03 PgRb1-22-21 PgRb1-15-02 PgRb1-17-10 PgRb1-16-04 PgRb1-18-02 PgRb1-21-02 PgRb1-12-01 PgRb1-13-01 PgCYP716A53v2_1 PgRb1-11-01 PgRb1-10-01
PgDS_1
PgRb1-09-22 PgSE2_4
Pgβ-AS_1 PgRb1-19-01 PgRb1-14-04
PgSS_1 PgCYP716A47_1 PgUGT71A27_2 PgCYP716A52v2_3
PgDS_3 PgSE2_1 PgFPS_22
S23 S21 S27 S14 S38 S7 S39 S6 S17 S5 S34 S11 S35 S1 S4 S24 S20 S42 S32 S18 S10 S40 S19 S12 S15 S36 S22 S31 S29 S26 S9 S3 S2 S41 S16 S25 S33 S28 S13 S37 S8 S30
c
cultivar
Figure S5. Expression heatmaps of the 21 PgRb1 genes with the 11 published PgRb1 genes at different developmental stages of plant roots (a), in different tissues of a four-year-old plant (b), and in the four-year-old roots of different cultivars (c).
